# Where we work correlates with whether we receive cardiorespiratory preventive care services: Health and Retirement Study 2003–2018

**DOI:** 10.1186/s12889-025-24748-z

**Published:** 2025-12-02

**Authors:** Wan-chin Kuo, Dian Luo, Roger L. Brown, Colleen Hruska, Chi-Jane Wang

**Affiliations:** 1 School of Nursing, University of Wisconsin-Madison, 701 Highland Avenue, Madison, WI 53705 USA; 2 Center for Demography of Health and Aging, University of Wisconsin-Madison, Madison, WI USA; 3 Department of Population Health Sciences, School of Medicine and Public Health, University of Wisconsin-Madison, Madison, WI USA; 4 Wisconsin State Association of Occupational Health Nurses, Reedsburg, WI USA; 5 Department of Nursing, College of Medicine, National Cheng Kung University, Tainan, Taiwan

**Keywords:** Cholesterol screening, Influenza vaccination, Health disparities, Preventive care services, Occupational health

## Abstract

**Background:**

Uncontrolled dyslipidemia contributes to cardiovascular diseases, the leading cause of death among American workers, while influenza leads to significant absenteeism and presenteeism. Despite the potential productivity loss due to cardiorespiratory illness, few studies have examined occupational disparities in preventive care utilization. This study aimed to assess the influence of occupation and job characteristics on cholesterol screening and influenza vaccination utilization.

**Methods:**

Data from the Health and Retirement Study (HRS) 2003–2018 were analyzed, representing past and current workers (*N* = 7,022). Occupation was coded based on the U.S. Census 1980 and 2000 and configured into five groups: management/science, social services, general services, health services, and industrial workforce. Participants who never reported job information were included in the sensitivity analysis. Job characteristics, including psychological and physical strains at work, ergonomic risk, lifting of heavy loads, and job stability, were based on self-reported questions. The influences of job category and job characteristics on cholesterol screening and influenza vaccination utilization were examined using multivariable logistic regression, multinomial logit model, and multinomial probit models.

**Results:**

Industrial workers and general services workers were less likely to receive cholesterol screenings and influenza vaccinations than management and science workers after controlling for social-demographic and health statuses. Frequent job-related heavy lifting and lower job-related mental strain were linked to lower cholesterol screening rates. Higher job-related physical strain and job instability were associated with reduced influenza vaccination uptake. Those outside the labor force were also less likely to undergo periodic cholesterol screening and influenza vaccination.

**Conclusion:**

This study demonstrated an underutilization of preventive care services among industrial and general services workers, as well as those outside the labor force. Our findings underscore the need to promote access to preventive care services while addressing cardiovascular disease awareness and vaccination hesitancy in these populations.

**Supplementary Information:**

The online version contains supplementary material available at 10.1186/s12889-025-24748-z.

## Introduction

Preventive care services are a critical aspect of public health practice, aimed at preventing conditions before they arise, catching conditions early, and maintaining health for individuals. These services encompass a range of activities, including screening for chronic conditions, cancer, and infectious diseases, as well as healthy lifestyle counseling. Among the commonly recommended preventive care services for adult populations are screening for cardiovascular risk factors, such as blood pressure and cholesterol checks, and annual influenza vaccination. In 2006, the American Heart Association (AHA) and American College of Cardiology (ACC) issued a consensus statement on secondary prevention for patients with coronary or other atherosclerotic cardiovascular disease (ASCVD) that recognized influenza vaccination as a Class 1B recommendation [1]. Mounting evidence has indicated the synergistic benefit of receiving cholesterol screening and influenza vaccination regularly, as the influenza vaccine significantly reduces the risk of cardiovascular and respiratory adverse outcomes, as well as a lower incidence of all-cause mortality [2, 3].

The AHA/ACC recommends that healthy adults, starting from the age of 20, establish the baseline and evaluate the 10-year ASCVD risk score every 4–6 years [4]. More frequent ASCVD risk assessments are recommended for those with chronic conditions or those aged above 50. Among the established ASCVD risk factors, dyslipidemia is an asymptomatic chronic condition characterized by states of reduced high-density lipoprotein cholesterol (HDL-C), elevations in low-density lipoprotein cholesterol (LDL-C), and triglycerides [5]. As an essential element of ASCVD risk assessment, the AHA/ACC recommends cholesterol screening every 4–6 years for adults without known ASCVD risk as an optimal frequency of screening. However, data from the National Health and Nutrition Examination Survey (NHANES, 2017–2018) shows that nearly 20% of adults in the U.S. have never had their cholesterol checked, and 27.5% did not meet the optimal screening recommendation (i.e., having not been screened in the past five years) [6]. There was a low rate of statin use (52.3%) among individuals with familial hypercholesterolemia, and an even lower rate among those with severe dyslipidemia (37.6%) [7]. Further, screening rates varied by racial and ethnic group, with the Hispanic and non-Hispanic Black groups being the least likely to ever have cholesterol screened [6]. On the other hand, influenza vaccination is recommended for adults annually as an optimal vaccination frequency [8]. Despite these recommendations, adults’ influenza vaccination uptake has decreased over the past ten years [9]. According to the Centers for Disease Control and Prevention (CDC), only 46.9% of adults aged 18 and older received influenza vaccination during the 2022–2023 flu season, showing a 2.5% decrease compared to the previous season [8]. The vaccine coverage rates between ethnic groups also varied, with the Hispanic and non-Hispanic Black groups less likely to have optimal influenza vaccination frequency than people of other races and ethnicities [8].

Unlike more invasive tests, such as cancer screenings requiring specialized equipment in medical facilities, both cholesterol screening and influenza vaccines can be administered outside hospitals and clinics. This flexibility allows for the delivery of preventive care services in community settings, local pharmacies, and workplaces, reducing barriers that might otherwise prevent individuals from accessing these services. Workplace cholesterol screenings and influenza vaccinations are particularly important for several reasons. First, influenza outbreaks in the workplace lead to absenteeism and presenteeism, both of which negatively impact productivity [10]. Moreover, these outbreaks can extend beyond the workplace, spreading flu to employees’ families and the broader community. By vaccinating a large portion of the workforce, businesses can reduce productivity losses due to respiratory illness while also contributing to public health by building herd immunity and protecting vulnerable populations with weaker immune systems [11]. Second, ASCVD is the leading cause of death and permanent disability among aging workers [12, 13]. Timely and effective cholesterol screening and management in the workplace can help employees adopt healthier lifestyles and reduce their risk of ASCVD. By addressing these risks early, employers can enhance their employees’ well-being and minimize productivity losses associated with cardiovascular diseases. Despite the lack of evidence to support the short-term return on investment (ROI), empirical studies have endorsed the long-term benefits of onsite medical clinics at the workplace [14].

Industrial and general services workers have long been linked to adverse health outcomes, with a disproportionally higher prevalence of ASCVD [15], metabolic syndrome [16], chronic bronchitis [17], and Chronic Obstructive Pulmonary Disease (COPD) [18]. The underlying mechanism of cardiometabolic health disparities in industrial and general services workers is multifactorial. Cumulative evidence indicates that industrial and general services workers experience a higher risk of occupational exposures (e.g., thermal, chemical, and mechanical risk) [19], unmet healthcare needs [20, 21], smoking or exposure to secondhand smoking [22], and long hours of monotonous/repetitive tasks, and ergonomic risk [19, 23, 24]. Emerging evidence further highlights the convolution of ASCVD and musculoskeletal injuries in industrial workers, indicating that higher levels of job-related physical strain, frequent lifting of heavy loads at work, and work-related musculoskeletal and ergonomic injuries were associated with higher levels of systolic blood pressure, total cholesterol, and ASCVD risk score [25, 26].

Despite empirical evidence highlighting the cardiorespiratory health disparities experienced by industrial and general services workers, these studies predominantly focus on health outcomes or hazard exposures. While several cross-sectional survey studies and qualitative studies reported unmet healthcare needs and underutilization of cholesterol screening and influenza vaccination in industrial and general services workers [27–29], to our knowledge, there is a lack of longitudinal evidence to support the role of occupational characteristics (e.g., job-related psychological strain, physical strain, or job stability) in the uptake of preventive care services. Hence, this study has two specific aims:

1. Examine the degree to which job categories are associated with the lower likelihood of receiving cholesterol screening and influenza vaccination.
2. Examine the correlations between job characteristics and periodical cholesterol screening and influenza vaccination.

## Methods

### Data source

**Fig. 1:**
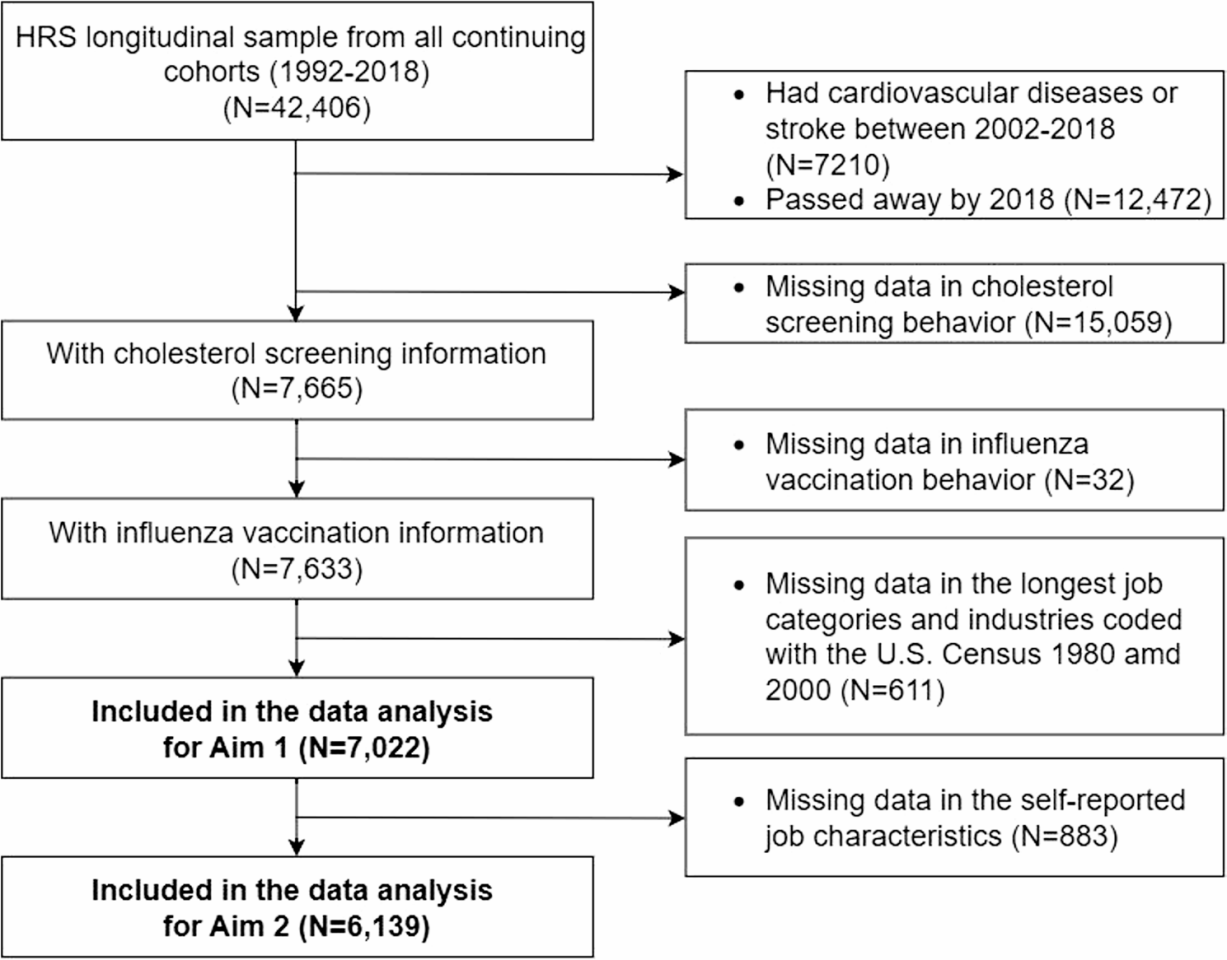
*The strengthening the reporting of observational studies in epidemiology (STROBE) diagram: final sample size in this study (Aim 1: N = 7,022; Aim 2: N = 6139)*

A longitudinal analysis was performed using the Health and Retirement Study (HRS) data. The HRS is a national longitudinal Survey of more than 37,000 individuals over age 50 in 23,000 U.S. households [30]. Since 1992, socioeconomic, psychological, and health data have been assessed in this core sample approximately every two years. Starting in 2006, half of this core sample was randomly assigned to enhanced face-to-face (EFTF) interviews with physical measures and a mail-back psychosocial questionnaire. Data from the EFTF interviews are available for every wave on half of the core sample and on the full sample every four years. Details about the design of the HRS are described elsewhere [30]. The current study focused on data collected from Wave 7 (HRS 2003–2004) to Wave 14 (HRS 2017–2018). Participants who passed away by the end of 2018, had CVD or stroke, had missing data in self-reported cholesterol screening and influenza vaccination, or without the U.S. Census 1980 or Census 2000 occupational codes reported in the longest job were excluded from the analysis [31]. In total, 7,022 participants were included in the analysis of Aim 1 (Fig. 1). Participants who did not report their Subjective job characteristics were excluded from the analysis of Aim 2, leaving a sample size of 6,139 for the analysis in Aim 2 (Fig. 1). The HRS was reviewed and approved by the University of Michigan’s Health Sciences Institutional Review Board (IRB). Participants were provided with a written informed consent document, read a confidentiality statement, and gave oral consent by agreeing to do the interview. This secondary data analysis was approved by the University of Wisconsin-Madison’s IRB with an exemption from full IRB review (ID: 2022 − 0517).

### Variables of interest

#### Outcomes of interest: preventive care utilization

C*holesterol screening behavior* was assessed by asking participants whether they had undergone any blood tests for cholesterol since the previous survey. Given the structure of the HRS survey—administered biannually with half the core sample and every four years with the full sample—cholesterol screening data were available approximately every four years. Therefore, longitudinal data from 16 years were consolidated into four assessment periods: 2003–2006, 2007–2010, 2011–2014, and 2015–2018. *Periodical cholesterol screening* was defined as having at least one cholesterol test during each four-year period across the 16 years. For each assessment period, participants were categorized as “yes” (1) or “no” (0) based on self-reported cholesterol screening. The cumulative frequency of cholesterol screenings was calculated as the sum of these responses, yielding a score from 0 (no screenings over 16 years) to 4 (received cholesterol screening(s) during each assessment period). Supplement 1 illustrates the operational definition of *periodical cholesterol screening*.

*Influenza vaccination behavior* was assessed by asking participants whether they had received any flu shots since the previous survey. Flu shot data, similar to cholesterol screening information, were collected approximately every four years over a 16-year period. The four assessment periods were 2003–2006, 2007–2010, 2011–2014, and 2015–2018. For each assessment period, participants were categorized as “yes” (1) or “no” (0) based on self-reported influenza vaccination. Periodical influenza vaccination was defined as receiving at least one flu shot during each four-year period across the 16 years. The cumulative frequency of influenza vaccination was calculated as the sum of these responses, yielding a score from 0 (never received flu shot over 16 years) to 4 (received flu shot(s) during each assessment period). Supplement 1 illustrates the operational definition of periodical influenza vaccination.

#### Exploratory variables: occupational categories and characteristics

Respondents’ longest-reported job titles were determined using retrospective job history data collected during participants’ initial interview upon entering the cohort study, as well as prospective job information obtained during each subsequent interview [32]. *The longest job category* was based on the participants’ longest reported job title coded by the U.S. Census 2000 occupational code and replaced with the reported job categories with the U.S. Census 1980 occupational code, U.S., Census 2002 industry code, or U.S. Census 1980 industry code, if participants’ U.S. Census 2000 code was missing [31]. The 25 occupational codes were further configured into five categories: (1) management/science, (2) social services, (3) general services, (4) health services, and (5) industrial workforce. The detailed U.S. Census 1980 and 2000 occupational codes for these five job categories are described in Supplement 2.

*Job characteristics*, including mental and physical strains at work, ergonomic risk, and Lifting heavy loads, were based on self-reported questions on a 4-point Likert scale (strongly agree, agree, disagree, or strongly disagree). Responses were re-coded as the higher score indicating a higher trait on that item [32].

1. Job-related physical strain: *My job requires lots of physical effort*.
2. Job-related psychological strain: *My job involves a lot of (psychological) stress*.
3. Job-related ergonomic risk: *My job requires stooping*,* kneeling*,* or crouching*.
4. Job-related heavy listing: *My job requires lifting heavy loads*.

Finally, job stability was defined by years of tenure at the longest-reported job. Longest job tenure was determined by comparing the durations from various sources: retrospective job histories, current job tenures reported in recent and previous interviews, and tenures for any jobs that ended between interviews up to the present. The longest non-missing tenure from these sources was identified as the longest job tenure [32].

#### Covariates

Covariates were identified based on the review of previous evidence for correlates of cholesterol screening and influenza vaccination behaviors. In total, thirteen covariates, including age, gender, race, educational attainment, income per capita, disability, number of private insurance, self-rated health, depression, smoking status, diagnosis of diabetes, diagnosis of hypertension, and body mass index (BMI), were identified as possible predictors of cholesterol screening and influenza vaccination behaviors. Household income per capita was used, which is the sum of household income received over the last calendar year, including business or farm income, self-employment earnings, gross rent, dividend and interest income, trust funds or royalties, and other asset income. Income was log-transformed to reduce the skewness and normalize the distribution following the recommendation from previous literature [33]. Disability was a binary indicator asking whether a respondent received or applied for disability benefits through Social Security Disability. Self-rated health was measured using a Likert-type scale asking respondents’ self-reported general health status, ranging from 1 (Excellent) to 5 (Poor). Depressive symptoms were evaluated based on the 8-item Center for Epidemiologic Studies Depression Scale (CES-D8), operationalized in a composite score based on a list of eight feelings, including low spirit, difficulty to do things, poor sleep, unhappiness, loneliness, life unsatisfaction, sadness, and life difficulty [34]. Gender, years of education, and race were treated as time-invariant covariates; age, income, health insurance, disability status, depression, smoking status, diagnosis of hypertension, diagnosis of diabetes, Body Mass Index (BMI), and self-rated health were treated as time-varying covariates and were based on the statuses reported at baseline wave (HRS Wave 7: 2003–2004).

### Statistical analysis

Sample characteristics were summarized using descriptive statistics. Missing values were identified in smoking, BMI, CES-D8, and the number of private insurance. The diagnosis of missing patterns with Little’s test for MCAR was not significant (X = 32.22; *p* = 0.096), supporting the assumption that the missing data was random. Hence, Multiple Imputation (MI) was performed using the Monte Carlo Markov Chain (MCMC) method based on 100 MCMC iterations with five imputed datasets using Mplus software [35].

The non-multicollinearity assumption was tested using the variance inflation factor (VIF) and tolerance value. The VIF ranged between 1.02 and 1.78, and the tolerance value ranged between 0.56 and 0.99, indicating that the non-multicollinearity assumption was met. Multivariable logistic regression was used to examine whether and the degree to which job categories and job characteristics affect the probability of periodical cholesterol screening and influenza vaccination (Yes/No) over 16 years.

The Score Test for the proportional odds assumption was conducted to assess whether the logit surfaces were parallel, a critical assumption of the ordered logistic model [36]. The test results indicated a significant violation of this assumption for both the cholesterol screening outcome (X^2^ = 726; DF = 18, p < 0.001) and influenza vaccination outcome (X^2^ = 1025; DF = 18, p < 0.001), indicating that the proportional odds assumption was not met. Therefore, the multinomial logit model was used, which treated the response as nominal (unordered) with frequency=’4’ (i.e., screened/vaccinated at every assessment period across the 16 years) as the reference category. In the sensitivity analysis, we followed Meyers’ approach by fitting multinomial probit models and comparing the results with multinomial logit models to see if the assumption of independence of irrelevant alternatives (IIA) held for our primary logit models [37]. Finally, we performed sensitivity analysis to examine whether excluding participants who never reported the longest job categories biases the estimates. Statistical analyses were performed using SAS software version 9.4 and STATA software version 18. All reported p-values were two-tailed, with p-values less than 0.05 considered significant.

## Results

### Sample characteristics

A total of 7,022 participants were included in the current study. As described in Table 1, the mean age at baseline was 60.9 years (SD = 8.4). Among the participants, 2,643 (37.6%) were men, and the racial composition was 5,610 (79.9%) White, 986 (14.0%) Black, 426 (6.1%) Other, and 5,090 (72.5%) Non-Hispanic White. Regarding educational attainment, 221 (3.2%) had less than 6th-grade education, 317 (4.5%) had 6–8 grade education, 2,925 (41.7%) had 9–12 grade education, 2,565 (36.5%) had a bachelor’s degree, and 994 (14.2%) had a graduate degree. Additionally, 5,301 (75.5%) participants were covered by private insurance. The annual income per capita was $17,250 (SD=$72,288). Compared to those with job information, participants without job information (*N* = 611) had disproportionately higher rates of disability (*p* < 0.001), diabetes (*p* = 0.001), hypertension (*p* < 0.001), depression (*p* < 0.001), and poor self-rated health (*p* < 0.001).

**Table 1:** Descriptive results of sample characteristics

	With job information (*N* = 7,022)		Without job information (*N* = 611)		*p*-value^d^
	Frequency (or mean)	% (or SD)	Frequency (or mean)	% (or SD)	
Social-Demographics					
Age, mean (SD)	60.9	8.4	65.8	10.5	**< 0.001**
Men, N (%)	2,643	37.6	138	22.6	**< 0.001**
Race, N (%)					0.214
White	5,610	79.9	480	78.6	
Black	986	14.0	84	13.8	
Other^a^	426	6.1	47	7.7	
Non-Hispanic White, N (%)	5,090	72.5	409	66.9	**0.003**
Education attainment, N (%)					**< 0.001**
<6 grade	221	3.2	54	8.84	
6–8 grade	317	4.5	63	10.31	
9–12 grade	2,925	41.7	277	45.34	
Bachelor’s degree	2,565	36.5	172	28.15	
Graduate degree	994	14.2	45	7.36	
Covered by private insurance (%)	5,301	75.5	251	41.08	**< 0.001**
Annual income per capita, mean (SD)	17,250	72,288	12,951	52,057	**< 0.001**
Occupational characteristics					
Longest job category, N (%)					
Management/Science	1,685	24.0	N/A	N/A	
Social services	596	8.5	N/A	N/A	
General services	2,768	39.4	N/A	N/A	
Health services	348	5.0	N/A	N/A	
Industrial workforce	1,625	23.1	N/A	N/A	
Job-related mental strain ^b^	1.5	0.9	N/A	N/A	
Job-related physical strain ^b^	1.1	1.1	N/A	N/A	
Job-related ergonomic risk ^b^	1.0	1.0	N/A	N/A	
Job-related lifting ^b^	0.6	0.9	N/A	N/A	
Tenure on the longest job (years) ^b^	20.8	11.2	N/A	N/A	
Health Statuses					
Disability, N (%)	203	2.9	40	6.6	**< 0.001**
Diagnosis of diabetes, N (%)	775	11.0	94	15.4	**0.001**
Diagnosis of hypertension, N (%)	2,831	40.3	294	48.1	**< 0.001**
BMI, mean (SD)	28.1	5.4	28.0	6.0	0.729
CES-D8, mean (SD)^c^	1.2	1.8	1.7	2.2	**< 0.001**
Cigarette smoking, N (%)	1,001	14.3	75	12.3	0.189
Self-rated health, N (%)					**< 0.001**
Excellent	1,285	18.3	69	11.29	
Very good	2,457	35.0	174	28.48	
Good	2,159	30.8	197	32.24	
Fair	899	12.8	114	18.66	
Poor	222	3.2	57	9.33	

*Abbreviation: SD *Standard deviation, *BMI *Body Mass Index

^a^Includes American Indian, Alaska Native, Asian or Pacific Islander

^b^On a 4-point Likert scale from 1 to 4, the higher the value, the higher degree the job characteristic was reported, based on the sample size of Aim 2 (*N* = 6,139)

^c^CES-D8, higher values indicate higher levels of depressive symptoms [range: 0–8]

^d^Normally distributed continuous variables were tested using the independent-sample T test; categorical variables were tested using the Mantel-Haenszel Chi-Square test; non-normally distributed continuous variables were tested using the Wilcoxon-Mann-Whitney test

**Bold font** indicates that the p-value was less than 0.05, two-tailed

### Job categories correlate with cholesterol screening and influenza vaccination

*Cholesterol Screening. *As shown in Table 2, although strong associations between job categories and periodical cholesterol screening were observed in the crude model, the adjustment of social-demographic and health statuses attenuates the magnitudes of these associations. Specifically, industrial workers (OR = 0.73, 95% CI=[0.62, 0.87], *p* < 0.001) and general services workers (OR = 0.78, 95% CI=[0.67, 0.90], *p* = 0.001) were less likely to receive periodical cholesterol screening compared to management and science workers, after the adjustment of social-demographic and health statuses. Supplement 3 illustrates the probability of cholesterol screening frequency across 16 years by five job categories (X axis). The five-color curves present the frequency of cholesterol screening during the four assessment periods (2003–2006, 2007–2010, 2011–2014, and 2015–2018). As noted in the purple curve, the general service (JOB = 3) and industrial workforce (JOB = 5) had a relatively lower probability of periodical cholesterol screening (i.e., screened cholesterol at all four assessment periods) compared to the other three job categories. Supplement 4 describes the detailed odds ratios by job categories, compared to the management and science workers, at various frequencies of cholesterol screening across the four assessment periods in the fully adjusted model.

**Table 2:** *Association between job categories and periodical utilization of preventive care services, including cholesterol screening and influenza vaccination (N = 7,022)*

	Periodical Cholesterol Screening (*N* = 7,022)								Periodical Influenza Vaccination (*N* = 7,022)							
Logistic Regression models	Crude Model				Fully Adjusted Model				Crude Model				Fully Adjusted Model			
Effect	OR	95% CI		p-value^b^	OR	95% CI		p-value^b^	OR	95% CI		P-value^b^	OR	95% CI		p-value^b^
Social Services	**0.75**	**0.61**	**0.91**	**0.004**	0.84	0.68	1.03	0.095	**0.73**	**0.60**	**0.88**	**0.001**	0.98	0.80	1.20	0.830
General Services	**0.64**	**0.56**	**0.73**	**< 0.001**	**0.78**	**0.67**	**0.90**	**0.001**	**0.67**	**0.59**	**0.76**	**< 0.001**	**0.86**	**0.75**	**0.99**	**0.038**
Health Services	**0.67**	**0.52**	**0.85**	**0.001**	0.89	0.69	1.16	0.390	**0.74**	**0.59**	**0.93**	**0.011**	1.26	0.98	1.62	0.077
Industrial Workforce	**0.53**	**0.46**	**0.61**	**< 0.001**	**0.73**	**0.62**	**0.87**	**< 0.001**	**0.54**	**0.47**	**0.62**	**< 0.001**	**0.82**	**0.69**	**0.97**	**0.023**
Management & Science	Ref.				Ref.				Ref.				Ref.			
Men					0.89	0.80	1.01	0.060					**0.79**	**0.70**	**0.88**	**< 0.001**
Women					Ref								Ref			
Education					**1.15**	**1.07**	**1.23**	**< 0.001**					**1.24**	**1.16**	**1.33**	**< 0.001**
Age					**1.02**	**1.01**	**1.03**	**< 0.001**					**1.08**	**1.07**	**1.09**	**< 0.001**
Black					**0.76**	**0.66**	**0.89**	**0.001**					**0.42**	**0.36**	**0.50**	**< 0.001**
Other^a^					0.86	0.70	1.07	0.181					**0.76**	**0.60**	**0.96**	**0.021**
White					Ref								Ref			
Insurance					**1.29**	**1.16**	**1.42**	**< 0.001**					**1.28**	**1.16**	**1.41**	**< 0.001**
Household income per cap					**1.04**	**1.03**	**1.06**	**< 0.001**					**1.03**	**1.02**	**1.04**	**< 0.001**
Disability					1.22	0.89	1.67	0.221					**1.76**	**1.29**	**2.40**	**< 0.001**
Without disability					Ref								Ref			
CES-D8					**0.95**	**0.92**	**0.97**	**< 0.001**					**0.94**	**0.91**	**0.97**	**< 0.001**
Smoking					**0.64**	**0.55**	**0.74**	**< 0.001**					**0.67**	**0.57**	**0.79**	**< 0.001**
Non-smoking					Ref								Ref			
DM					**1.82**	**1.50**	**2.20**	**< 0.001**					**1.63**	**1.37**	**1.93**	**< 0.001**
Without DM					Ref								Ref			
HTN					**2.10**	**1.88**	**2.36**	**< 0.001**					**1.42**	**1.27**	**1.59**	**< 0.001**
Without NTN					Ref								Ref			
BMI					1.01	1.00	1.02	0.158					1.00	0.99	1.01	0.727
SRH					**1.08**	**1.02**	**1.15**	**0.012**					**1.09**	**1.02**	**1.16**	**0.006**

*Abbreviation*: *OR *Odds Ratio, *95% CI *95% Confidence Interval, *CES-D8 *Center for Epidemiologic Studies Depression Scale-8 Items, *DM *Diabetes Mellitus,* HTN *Hypertension, *BMI *Body Mass Index, *SRH *Self-Rated Health

^a^Includes American Indian, Alaska Native, Asian or Pacific Islander

^b^**Bold font** indicates that the p-value was less than 0.05, two-tailed

*Influenza Vaccination*. Again, the strong associations between job categories and periodical influenza vaccination observed in the crude model were attenuated by the adjustment of social-demographic and health statuses. As shown in the fully adjusted model (Table 2), industrial workers (OR = 0.82, 95% CI=[0.69, 0.97], *p* = 0.023) and general services workers (OR = 0.86, 95% CI= [0.75, 0.99], *p* = 0.038) were less likely to receive periodical influenza vaccination compared to management and science workers. Supplement 5 illustrates the probability of influenza vaccination frequency across 16 years by five job categories (X axis). Again, similar to what we found in cholesterol screening patterns, as illustrated in the purple curve, the general service (JOB = 3) and industrial workforce (JOB = 5) had a relatively lower probability of periodical influenza vaccination (i.e., vaccinated at all four assessment periods) compared to the other three job categories. Supplement 4 describes the detailed odds ratios by job categories, compared to the management and science workers, at various frequencies of influenza vaccination across the four assessment periods in the fully adjusted model.

### Job characteristics correlate with cholesterol screening and influenza vaccination

As shown in Table 3, higher job-related mental strain was associated with an increased likelihood of periodic cholesterol screening (OR = 1.10, 95% CI [1.03, 1.18], *p* = 0.005), while higher job-related lifting was associated with a lower likelihood (OR = 0.90, 95% CI [0.83, 0.97], *p* = 0.009), after adjusting for health status and sociodemographic factors. Conversely, higher job-related physical strain was associated with a lower likelihood of periodic influenza vaccination (OR = 0.88, 95% CI [0.83, 0.95], *p* < 0.001), with job stability showing a marginal association (OR = 1.01, 95% CI [1.00, 1.01], *p* = 0.011). Job-related ergonomic risk was not associated with either periodic cholesterol screening or influenza vaccination.

**Table 3:** *Association between job characteristics and periodical utilization of preventive care services, including cholesterol screening and influenza vaccination, controlled for the social-demographic and health statuses (N = 6,139)*

	Cholesterol Screening (*N* = 6,139)								Influenza Vaccination (*N* = 6,139)							
	Crude Model				Fully Adjusted Model				Crude Model				Fully Adjusted Model			
Effect	OR	95% CI		p-value^b^	OR	95% CI		p-value^b^	OR	95% CI		P-value^b^	OR	95% CI		p-value^b^
Longest job (years)	**1.01**	**1.01**	**1.02**	**< 0.001**	1.01	1.00	1.01	0.071	**1.01**	**1.01**	**1.02**	**< 0.001**	**1.01**	**1.00**	**1.01**	**0.011**
Job-related lifting	**0.86**	**0.79**	**0.93**	**< 0.001**	**0.90**	**0.83**	**0.97**	**0.009**	**0.91**	**0.84**	**0.99**	**0.025**	0.97	0.89	1.06	0.501
Job-related mental strain	**1.09**	**1.03**	**1.16**	**0.005**	**1.10**	**1.03**	**1.18**	**0.005**	1.00	0.94	1.07	0.927	1.04	0.97	1.11	0.259
Job-related ergonomic risk	0.95	0.89	1.02	0.139	1.01	0.94	1.08	0.858	0.94	0.88	1.01	0.070	1.00	0.93	1.07	0.879
Job-related physical strain	**0.92**	**0.87**	**0.98**	**0.013**	0.95	0.89	1.01	0.113	**0.87**	**0.81**	**0.92**	**< 0.001**	**0.88**	**0.83**	**0.95**	**< 0.001**
Men					**0.84**	**0.75**	**0.95**	**0.005**					**0.74**	**0.66**	**0.83**	**< 0.001**
Women					Ref								Ref			
Education					**1.16**	**1.09**	**1.25**	**< 0.001**					**1.24**	**1.15**	**1.33**	**< 0.001**
Age					**1.03**	**1.02**	**1.03**	**< 0.001**					**1.08**	**1.07**	**1.08**	**< 0.001**
Black					**0.75**	**0.64**	**0.89**	**0.001**					**0.43**	**0.36**	**0.51**	**< 0.001**
Other^a^					0.92	0.73	1.16	0.465					0.90	0.70	1.15	0.382
White					Ref								Ref			
Insurance					**1.35**	**1.21**	**1.51**	**< 0.001**					**1.27**	**1.14**	**1.42**	**< 0.001**
Household income per cap					**1.04**	**1.02**	**1.05**	**< 0.001**					**1.02**	**1.01**	**1.04**	**0.003**
Disability					1.11	0.72	1.70	0.643					**1.60**	**1.05**	**2.45**	**0.030**
Without disability					Ref								Ref			
CES-D8					**0.94**	**0.90**	**0.97**	**< 0.001**					**0.95**	**0.91**	**0.98**	**0.002**
Smoking					**0.65**	**0.56**	**0.76**	**< 0.001**					**0.69**	**0.58**	**0.83**	**< 0.001**
Non-smoking					Ref								Ref			
DM					**1.82**	**1.47**	**2.24**	**< 0.001**					**1.73**	**1.44**	**2.09**	**< 0.001**
Without DM					Ref								Ref			
HTN					**2.15**	**1.90**	**2.44**	**< 0.001**					**1.40**	**1.24**	**1.58**	**< 0.001**
Without NTN					Ref								Ref			
BMI					1.01	1.00	1.02	0.052					1.00	0.99	1.01	0.719
SRH					**1.08**	**1.01**	**1.15**	**0.021**					**1.08**	**1.01**	**1.15**	**0.029**

*Abbreviation:OR *Odds Ratio,* 95% CI *95% Confidence Interval,* (c) CES-D8 *Center for Epidemiologic Studies Depression Scale-8 Items,* DM *Diabetes Mellitus,* HTN *Hypertension,* BMI *Body Mass Index*, SRH *Self-Rated Health

^a^Includes American Indian, Alaska Native, Asian or Pacific Islander

^b^**Bold font** indicates that the p-value was less than 0.05, two-tailed

### Sensitivity analysis

We fitted multinomial probit models and compared the results with multinomial logit models for sensitivity analysis. As shown in Supplement 4, the directionality and significance of all coefficients are consistent between the multinomial probit and multinomial logit models, supporting the robustness of the point estimates.

To assess the potential bias of excluding participants who never reported the longest job categories, we conducted a sensitivity analysis by including these participants in the fully adjusted models. As shown in Supplement 7 and Supplement 8, the effects we observed in industrial workers remained significant, with no change in directionality or significance, after including those who never reported the longest job categories (Job = 0). However, we found that those who did not report job categories (Job = 0) were also less likely to undergo periodic cholesterol screening and influenza vaccination.

## Discussion

Using longitudinal nationwide data from the HRS 2003–2018, we demonstrated the occupational correlates of cardiorespiratory preventive care utilization. In particular, industrial, general services workers, as well as those outside the labor force (either intentional or unintentional), had a lower likelihood of periodic cholesterol screening and influenza vaccination compared to management and science workers as they transitioned into mid to late life.

We found that workers with higher levels of job-related heavy lifting and physical strain were associated with a lower likelihood of optimal cholesterol screening and influenza vaccination. Toward this finding, we speculated that viewing job-related heavy lifting and physical strain as “workouts” might lead to overestimating compliance with current physical activity and fitness recommendations (i.e., 150 min of moderate-intensity activity, or 75 min of vigorous-intensity activity per week), thereby underestimating the importance of engaging in preventive cardiorespiratory care [38]. For instance, Arias et al. reported that two-thirds of construction workers’ daily activity patterns are occupational physical activity (OPA), leaving only about 10 min of leisure-time physical activity per day [39]. While more evidence is warranted to confirm our speculation, this observation underscores the need to increase awareness of the *physical activity paradox*, in which prolonged OPA does not equate to better cardiometabolic outcomes [40, 41]. Cardiorespiratory fitness requires intermittent, high-intensity bouts (> 60–80% of maximal aerobic capacity) with recovery [42], whereas typical OPA involves repetitive tasks at only 30–35% capacity over an 8-hour day [43].

Despite the evidence linking higher psychological stress to worsened health behaviors and metabolic profile [44, 45], the notion that “occupational stress might hinder the uptake of preventive care services” was not supported by the current analysis. Instead, higher occupational stress, in the current study, was associated with a higher likelihood of optimal cholesterol screening. It is possible that workers who are aware of their occupational stress are more likely to utilize preventive care services to protect their health [46]. Yet, reverse causation, in which behavior often influences attitudes in the real world, might confound our findings, leading to the spurious result [47]. In other words, workers who prioritize self-care might become more aware of their occupational stress. However, caution should be taken when interpreting this finding, because the concept of “occupational stress” could be endorsed differently by workers across occupational sectors or skilled trades. A single question, in the current study, limits our interpretation of workers’ sources, domains, and context of occupational stress [48, 49]. While traditional instruments, such as Demand-Control or Effort-Reward Imbalance models, were predominantly used, more novel instruments, such as the Job Demands-Resources Model, might offer fresh insights into job demands and resources in modern post-industrial society [50].

Our findings corroborate previous literature regarding preventive healthcare disparities in industrial and general services workers [21, 27, 28, 51]. For instance, according to the National Survey of Long-Haul Truck Driver Health and Injury, about 80% of truck drivers don’t receive influenza vaccination annually, and 25.2% of male drivers and 34.4% of female drivers did not have their cholesterol checked [27]. In terms of why preventive care services were underutilized in these populations, it is possible that workers avoid regular check-ups or screenings due to concerns about missing work and out-of-pocket expenses. For instance, a previous survey indicated that the primary reasons industrial employees received the influenza vaccine at the workplace were economic concerns (free 84%; convenient 80%; avoid absenteeism 82%) rather than health-related concerns [52]. However, a more pressing concern might be the lack of access or potential out-of-pocket costs. For instance, in 2014, only 14.4% of restaurant workers had employer-based health insurance, compared to the national average of 41.9% [28]. Apostolopoulos and colleagues found that 33% of truck drivers had no health insurance, 70% had no regular healthcare visits, 24.4% could not afford insurance, and 42.1% took over-the-counter drugs when sick [53]. As such, workers might opt for high-deductible plans, limit interactions with healthcare providers, reduce doctor visits, and change physicians annually [54]. Even during the preventive health visits for the renewal of a commercial driver’s license (CDL), as Goldstein reflected in his case study [54], the truck driver said, “*I know the longer I talk*,* the more I tell you*,* the more I explain*,* the higher your bill*.” These workers have fewer opportunities to make informed decisions about the benefits of influenza vaccination, cholesterol management, or recommendations from the CDC and AHA, leaving them at a higher risk for preventable health issues [21].

Finally, by focusing on the working population, our original hypothesis overlooked the profound health disparities existing outside the labor force, including those who were unemployed, non-wage workers, homemakers, or unpaid caregivers. As illustrated in Table 1, participants without job information had disproportionately higher rates of disability, diabetes, hypertension, depression, and poor self-rated health. Furthermore, our longitudinal data analysis does add to the literature by showing a relatively lower likelihood of preventive care utilization in this group, after controlling for these confounders [55]. Addressing the health disparities in unemployed groups (whether due to intentional or unintentional factors, non-wage workers, unpaid caregivers, or homemakers) requires greater community and public health support. A multi-faceted approach, in collaboration with public health agencies (e.g., CDC), is needed to reduce disparities and promote preventive care services in these populations [56].

### Implication to policy and practice

Where we work correlates with whether we receive preventive care services. Our findings raise a critical question: “How can we act on these disparities?” Viewing this as an opportunity to promote cardiorespiratory care services, we advocate for the promotion of workplace preventive care, given the underutilization of these services among industrial and service workers [57]. Our subsequent analysis of job characteristics provides insights explaining the impacts of job-related heavy lifting and physical strain on workers’ cardiorespiratory health. The traditional view of OPA as “workouts” may give workers a false sense of meeting physical activity guidelines and cardiorespiratory fitness, thereby potentially underestimating the need for preventive cardiorespiratory care [40, 41]. Furthermore, repetitive lifting or manual handling tasks are often accompanied by prolonged sitting or standing, which synergistically contribute to a higher risk of cardiovascular diseases, fatigue, and low back pain [24]. Occupational health clinicians and nurses play a crucial role in providing health education to increase cardiovascular disease awareness and reduce vaccination hesitancy, encouraging workers to seek these preventive services. It is also essential to explain the physical activity paradox to workers and encourage them to engage in leisure-time physical activity outside of work hours, while emphasizing the importance of ergonomic protection [38].

### Limitations

We recognize several limitations to this study. First, the dataset does not include younger working populations, limiting the generalizability of our findings to younger adults. Second, as job categories and characteristics evolve over time, our quantification of job characteristics and occupational categorization was based on participants’ longest-reported jobs and the characteristics identified in their most recent survey responses. This approach does not account for potential upward mobility in career paths, which may introduce bias in estimating the associations observed. Third, although longitudinal data were used to assess cholesterol screening and influenza vaccination from 2003 to 2018, the operational definitions of frequency and periodical screening were constrained by the cohort design, which measured these outcomes every four years. Hence, our definitions of periodical cholesterol screening and influenza vaccination may not fully align with current clinical guidelines. Fourth, job characteristics were derived from single-item, self-reported questions without objectivity and dimensionality. Caution should be taken when interpreting our correlational results between job characteristics and preventive health disparities due to the lack of dimensionality and objectivity in the criteria used to characterize working conditions [48, 58]. Finally, we have excluded participants with pre-existing cardiovascular conditions from the current analysis, as this group typically requires more intensive cholesterol management and faces higher risks of influenza complications, which subsequently influence their preventive care utilization [4]. Hence, the selection bias precludes the generalizability of our results to tertiary prevention.

## Conclusion

The 16-year data from the HRS reveal that industrial, general services workers, and those outside the labor force underutilize cholesterol screening and influenza vaccination. Our findings underscore the need to promote access to preventive care services while addressing cardiovascular disease awareness and vaccination hesitancy in these groups. Furthermore, our observation that workers’ job-related heavy lifting and physical strain are associated with lower preventive care utilization underscores the need for health education on the physical activity paradox and ergonomic protection.

## Supplementary Information

Supplementary Material 1.

## Data Availability

Health and Retirement Study (HRS) data were collected, managed, and maintained by the University of Michigan with funding from the National Institute on Aging (grant number U01AG009740), Ann Arbor, MI. Program code supporting the findings of this study are available from the corresponding author on request. All users who analyze HRS data should follow the HRS Data Access User Agreement.

## Abbreviations

SD: Standard deviation
BMI: Body Mass Index
