# Supplementary material for: Where we work correlates with whether we receive cardiorespiratory preventive care services: Health and Retirement Study 2003–2018
Source: BMC Public Health. 2025 Dec 2;26:69. doi: 10.1186/s12889-025-24748-z (PMC12777210; doi:10.1186/s12889-025-24748-z)
Supplement: Supplementary file 1 — Supplementary Material 1. [file 12889_2025_24748_MOESM1_ESM.docx]

**Supplemental Materials**

**Supplement 1.** *Operation of periodical cholesterol screening and influenza vaccination*

|  | Health and Retirement Study | | | | | | | |
| --- | --- | --- | --- | --- | --- | --- | --- | --- |
| HRS Study Wave (W) | W7 (2004) | W8 (2006) | W9 (2008) | W10 (2010) | W11 (2012) | W12 (2014) | W13 (2016) | W14 (2018) |
| Half sample | 2003-2004 | 2005-2006 | 2007-2008 | 2009-2010 | 2011-2012 | 2013-2014 | 2015-2016 | 2017-2018 |
| Full sample | 2003-2006 | | 2007-2010 | | 2011-2014 | | 2015-2018 | |
| Received cholesterol screening or not | Yes/No | | Yes/No | | Yes/No | | Yes/No | |
| Received influenza vaccination or not | Yes/No | | Yes/No | | Yes/No | | Yes/No | |
| Assessment periods | I | | II | | III | | IV | |
| Summation of cholesterol screening behaviors across four assessment periods | **Outcome Variable I: Cholesterol screening across 16 years**  0: no screenings over 16 years  1: reporting one yes (screened cholesterol at one assessment period)  2: reporting two yes (screened cholesterol at two assessment periods)  3: reporting three yes (screened cholesterol at three assessment periods)  4: reporting four yes (screened cholesterol at all assessment periods) | | | | | | | |
| Summation of influenza vaccination behaviors across four assessment periods | **Outcome Variable II: Influenza vaccination across 16 years**  0: no screenings over 16 years  1: reporting one yes (received influenza vaccination at one assessment period)  2: reporting two yes (received influenza vaccination at two assessment periods)  3: reporting three yes (received influenza vaccination at three assessment periods)  4: reporting four yes (received influenza vaccination at all assessment periods) | | | | | | | |
| Periodical cholesterol screening | **Outcome Variable III: Periodical cholesterol screening**  Yes: screened cholesterol at all assessment periods  No: did not receive cholesterol screening in at least one assessment period | | | | | | | |
| Periodical influenza vaccination | **Outcome Variable IV: Periodical influenza vaccination**  Yes: received influenza vaccination at all assessment periods  No: did not receive influenza vaccination in at least one assessment period | | | | | | | |

**Supplement 2.** *Operation of self-reported longest job categories based on the occupation codes in U.S. Census 2000 and 1980.*

| **Job Categories** | **U.S. Census 1980 Occupational Code^a^** | **U.S. Census 2000 Occupational Code ^b^** |
| --- | --- | --- |
| **Management & Science** | Managerial specialty operation | Management |
|  | Professional, Scientific, and Technical | Business Operations Specialists |
|  | Finance and Insurance | Financial Specialists |
|  | Real Estate and Rental and Leasing | Computer and Mathematical |
|  |  | Architecture and Engineering |
|  |  | Life, Physical, and Social Science |
| **Social Services** | Protection Services | Community and Social Services |
|  | Member of Armed Forces | Legal Occupations |
|  | Public Administration | Education, Training, and Library |
|  | Arts, Entertainment, and Recreation | Arts, Design, Entertainment, Sports, and Media |
|  | Educational Services | Protective Service |
|  | Information Services | Military Specific Occupations |
| **General Services** | Accommodations and Food Services | Food Preparation and Serving Occupations |
|  | Private Household, Cleaning and Building Services | Building and Grounds Cleaning and Maintenance |
|  | Personal Services | Personal Care and Service Occupations |
|  | Sales | Sales |
|  | Management, Administrative Support, and Waste | Office and Administrative Support |
| **Health Services** | Health services | Healthcare Practitioners and Technical Occupations |
|  | Health Care and Social Assistance | Healthcare Support Occupations |
| **Industrial Workforce** | Farming, forestry, fishing | Farming, Fishing, and Forestry |
|  | Construction trade and extractors | Construction Trades |
|  | Operators: machine | Extraction Workers |
|  | Mechanics and repair | Installation, Maintenance, and Repair Workers |
|  | Precision production | Production Occupations |
|  | Operators: transport, etc. | Transportation and Material Moving |
|  | Operators: handlers, etc. |  |

**^a^** U.S. Census 1980 Industry Code and U.S. Census 2002 Industry Code were used to supplement U.S. Census 1980 Occupational Code, due to fewer occupational categories in U.S. Census 1980.

**^b^** The longest job category was based on the participants’ longest reported job title coded by the U.S. Census 2000 occupational code and replaced with the reported job categories with the U.S. Census 1980 occupational code, U.S., Census 2002 industry code, or U.S. Census 1980 industry code, if participants’ U.S. Census 2000 code was missing.

**Supplement 3.** *Marginal probability of cholesterol screening frequency across the four assessment periods by job categories: (A) crude model results and (B) controlled for the social-demographic and health statuses.* **Note.** Job category (JOB): management & science=1; social services=2; general services=3; health services=4; industrial workforce=5


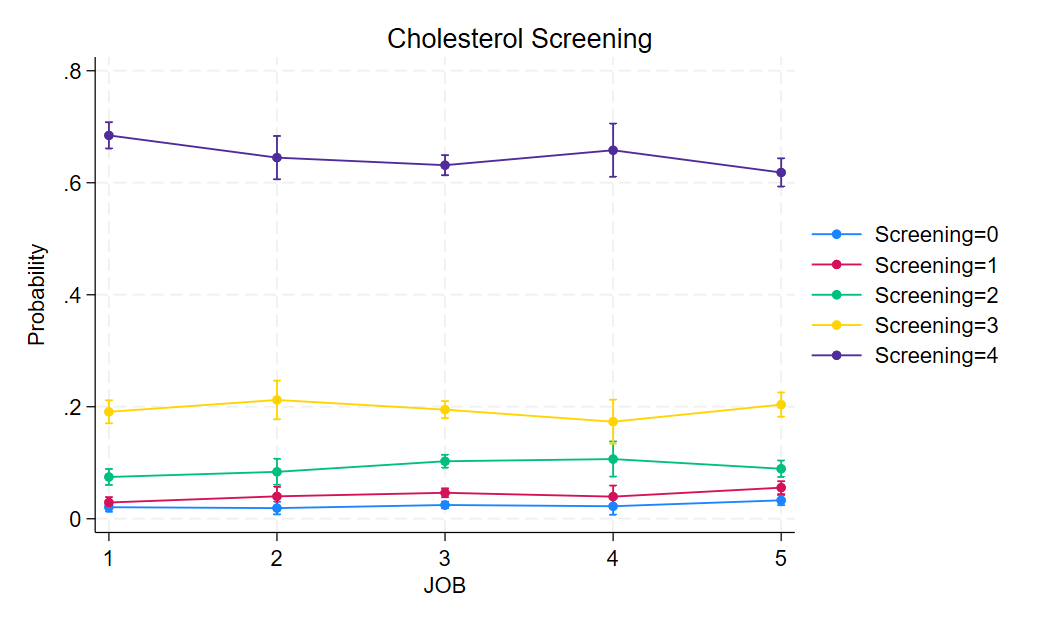

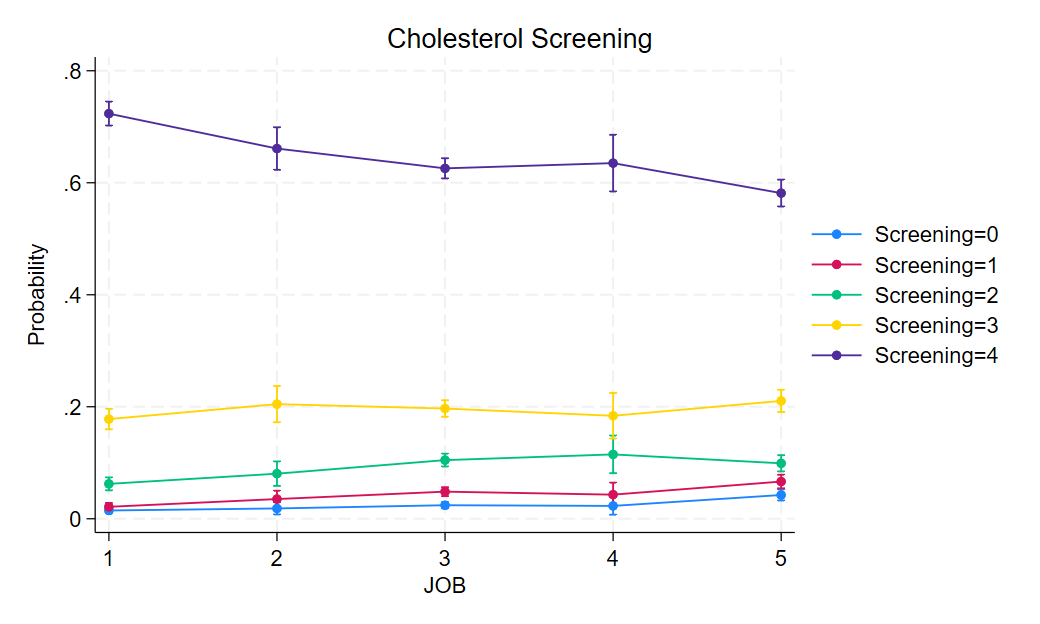


1. **Crude Model**
2. **Controlled for Social Demographics and Health Statuses**

**Supplement 4.** *Associations between job categories and the frequency of cholesterol screening and influenza vaccination across the four assessment periods, controlled for the social demographics and health statuses.*

|  |  | Cholesterol Screening (N=7,022) | | | | |  | Influenza Vaccination (N=7,022) | | | | |
| --- | --- | --- | --- | --- | --- | --- | --- | --- | --- | --- | --- | --- |
|  |  | Fully adjusted model **^c^** | | | | |  | Fully adjusted model **^c^** | | | | |
|  | Freq **^b^** | β | p-value | OR | OR 95% CI | |  | β | p-value | OR | OR 95% CI | |
| Social Services | 0 | 0.01 | 0.977 | 1.01 | 0.49 | 2.11 |  | -0.02 | 0.881 | 0.98 | 0.74 | 1.30 |
|  | 1 | 0.41 | 0.156 | 1.50 | 0.86 | 2.63 |  | -0.12 | 0.482 | 0.89 | 0.63 | 1.24 |
|  | 2 | 0.19 | 0.301 | 1.21 | 0.84 | 1.75 |  | 0.07 | 0.697 | 1.07 | 0.77 | 1.48 |
|  | 3 | 0.18 | 0.162 | 1.19 | 0.93 | 1.52 |  | 0.13 | 0.374 | 1.13 | 0.86 | 1.49 |
|  | 4 | Ref |  |  |  |  |  | Ref |  |  |  |  |
| General Services | 0 | 0.32 | 0.208 | 1.38 | 0.84 | 2.28 |  | **0.26** | **0.009** | **1.30** | **1.07** | **1.58** |
|  | 1 | **0.60** | **0.003** | **1.82** | **1.22** | **2.71** |  | -0.02 | 0.848 | 0.98 | 0.77 | 1.24 |
|  | 2 | **0.43** | **<.001** | **1.54** | **1.20** | **1.99** |  | **0.25** | **0.034** | **1.28** | **1.02** | **1.61** |
|  | 3 | 0.12 | 0.185 | 1.13 | 0.95 | 1.34 |  | 0.08 | 0.435 | 1.08 | 0.89 | 1.32 |
|  | 4 | Ref |  |  |  |  |  | Ref |  |  |  |  |
| Health Services | 0 | 0.17 | 0.702 | 1.18 | 0.51 | 2.74 |  | -0.27 | 0.141 | 0.77 | 0.54 | 1.09 |
|  | 1 | 0.38 | 0.247 | 1.46 | 0.77 | 2.78 |  | **-0.67** | **0.004** | **0.51** | **0.32** | **0.81** |
|  | 2 | **0.42** | **0.045** | **1.52** | **1.01** | **2.29** |  | -0.10 | 0.607 | 0.90 | 0.61 | 1.34 |
|  | 3 | -0.05 | 0.770 | 0.95 | 0.70 | 1.31 |  | -0.05 | 0.789 | 0.96 | 0.68 | 1.34 |
|  | 4 | Ref |  |  |  |  |  | Ref |  |  |  |  |
| Industrial Workforce | 0 | **0.66** | **0.015** | **1.94** | **1.14** | **3.31** |  | **0.26** | **0.028** | **1.30** | **1.03** | **1.65** |
|  | 1 | **0.82** | **<.001** | **2.26** | **1.46** | **3.49** |  | -0.01 | 0.930 | 0.99 | 0.75 | 1.30 |
|  | 2 | **0.33** | **0.033** | **1.38** | **1.03** | **1.87** |  | **0.33** | **0.016** | **1.39** | **1.06** | **1.82** |
|  | 3 | 0.19 | 0.072 | 1.21 | 0.98 | 1.48 |  | 0.18 | 0.126 | 1.20 | 0.95 | 1.52 |
|  | 4 | Ref |  |  |  |  |  | Ref |  |  |  |  |

**^a^** Reference for job category: Management & Science

**^b^** Reference for frequency of screening and vaccination: '4'

**^c^** Fully adjust model: Models were adjusted for age, gender, race, educational attainment, income per capita, disability, number of insurance, self-rated health, depression, smoking status, diagnosis of diabetes, diagnosis of hypertension, and body mass index

**Supplement 5.** *Marginal probability of influenza vaccination frequency across the four assessment periods by job categories: (A) crude model results and (B) controlled for the social-demographic and health statuses.* **Note.** Job category (JOB): management & science=1; social services=2; general services=3; health services=4; industrial workforce=5

1. **Crude Model Results**

**
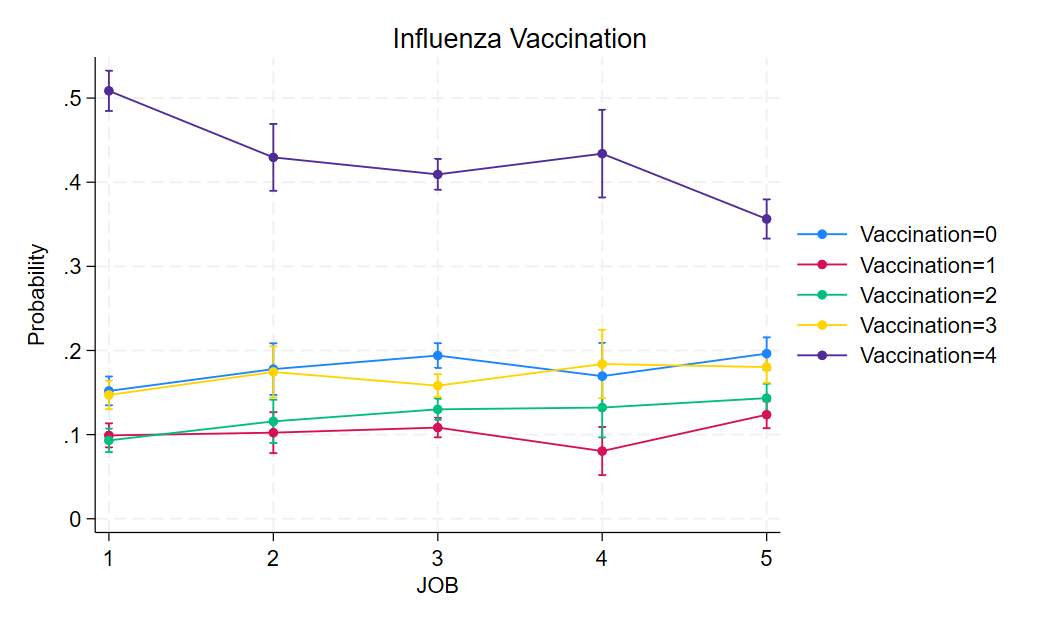
**

**(B) Controlled for Social Demographics and Health Statuses**

**
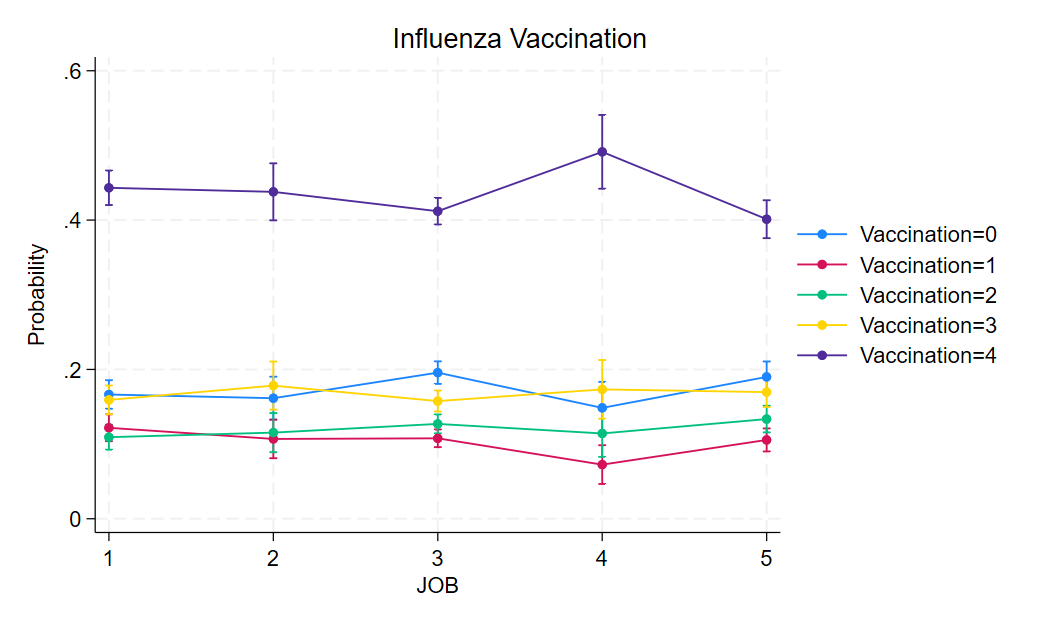
**

**Supplement 6.** *Sensitivity Analysis: Comparison of the results between multinomial logit and multinomial probit models*

|  |  | Cholesterol Screening (N=7,022) | | | | |  | Influenza Vaccination (N=7,022) | | | | |
| --- | --- | --- | --- | --- | --- | --- | --- | --- | --- | --- | --- | --- |
|  |  | Logit | |  | Probit | |  | Logit | |  | Probit | |
|  |  | β | p-value |  | β | p-value |  | β | p-value |  | β | p-value |
| Freq=0 | [Job=1] | Ref |  |  | Ref |  |  | Ref |  |  | Ref |  |
|  | [Job=2] | 0.01 | 0.98 |  | 0.03 | 0.88 |  | -0.02 | 0.88 |  | -0.01 | 0.96 |
|  | [Job=3] | 0.32 | 0.21 |  | 0.17 | 0.13 |  | **0.26** | **0.01** |  | **0.20** | **0.01** |
|  | [Job=4] | 0.17 | 0.70 |  | 0.07 | 0.73 |  | -0.27 | 0.14 |  | -0.19 | 0.15 |
|  | [Job=5] | **0.66** | **0.02** |  | **0.30** | **0.02** |  | **0.26** | **0.03** |  | **0.19** | **0.02** |
| Freq=1 | [Job=1] | Ref |  |  |  |  |  | Ref |  |  |  |  |
|  | [Job=2] | 0.41 | 0.16 |  | 0.18 | 0.20 |  | -0.12 | 0.48 |  | -0.07 | 0.57 |
|  | [Job=3] | **0.60** | **<0.01** |  | **0.25** | **0.01** |  | -0.02 | 0.85 |  | 0.01 | 0.87 |
|  | [Job=4] | 0.38 | 0.25 |  | 0.17 | 0.29 |  | **-0.67** | **<0.01** |  | **-0.41** | **0.01** |
|  | [Job=5] | **0.82** | **<.001** |  | **0.35** | **0.00** |  | -0.01 | 0.93 |  | 0.01 | 0.88 |
| Freq=2 | [Job=1] | Ref |  |  |  |  |  | Ref |  |  |  |  |
|  | [Job=2] | 0.19 | 0.30 |  | 0.10 | 0.33 |  | 0.07 | 0.70 |  | 0.05 | 0.65 |
|  | [Job=3] | **0.43** | **<.001** |  | **0.22** | **<0.01** |  | **0.25** | **0.03** |  | **0.17** | **0.03** |
|  | [Job=4] | 0.42 | 0.05 |  | 0.19 | 0.11 |  | -0.10 | 0.61 |  | -0.09 | 0.53 |
|  | [Job=5] | **0.33** | **0.03** |  | **0.17** | **0.04** |  | **0.33** | **0.02** |  | **0.23** | **0.01** |
| Freq=3 | [Job=1] | Ref |  |  |  |  |  | Ref |  |  |  |  |
|  | [Job=2] | 0.18 | 0.16 |  | 0.10 | 0.22 |  | 0.13 | 0.37 |  | 0.10 | 0.33 |
|  | [Job=3] | 0.12 | 0.19 |  | 0.07 | 0.20 |  | 0.08 | 0.44 |  | 0.07 | 0.33 |
|  | [Job=4] | -0.05 | 0.77 |  | -0.02 | 0.84 |  | -0.05 | 0.79 |  | -0.04 | 0.74 |
|  | [Job=5] | 0.19 | 0.07 |  | 0.11 | 0.10 |  | 0.18 | 0.13 |  | 0.14 | 0.10 |

**^a^** Frequency='4' as the reference category.

**^b^** Job Category (JOB): Management & Science=1; Social Services=2; General Services=3; Health Services=4; Industrial workforce=5.

**^c^** Models were controlled for age, gender, race, educational attainment, income per capita, disability, number of insurance, self-rated health, depression, smoking status, diagnosis of diabetes, diagnosis of hypertension, and body mass index (BMI).

**Supplement 7.** *Sensitivity analysis: Correlates of periodical cholesterol screening in the full sample (N=7,633), including those with and without job categories.*


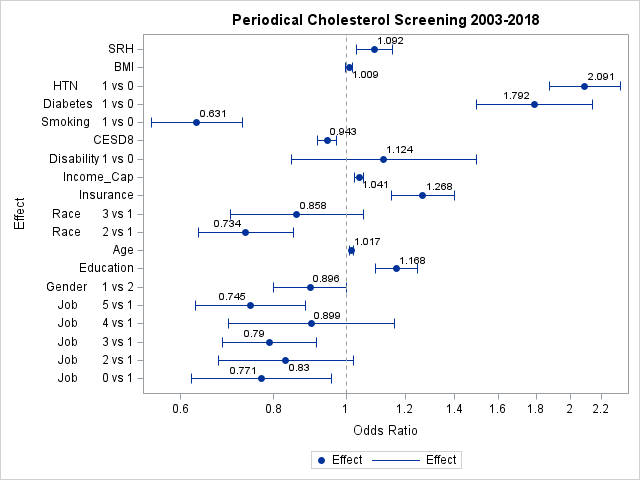


***Note.*** SRH=Self-rated health; BMI=Body mass index; HTN=hypertension; CES-D8=Center for Epidemiologic Studies Depression Scale-8 Items; Income_Cap=Household income per capita; Race: Others=3; Black=2. Gencer: men=1; women=2. Job Category (JOB): Management & Science=1; Social Services=2; General Services=3; Health Services=4; Industrial Workforce=5; Did not report job category=0.

**Supplement 8.** *Sensitivity analysis: Correlates of periodical influenza vaccination in the full sample (N=7,633), including those with and without job categories.*


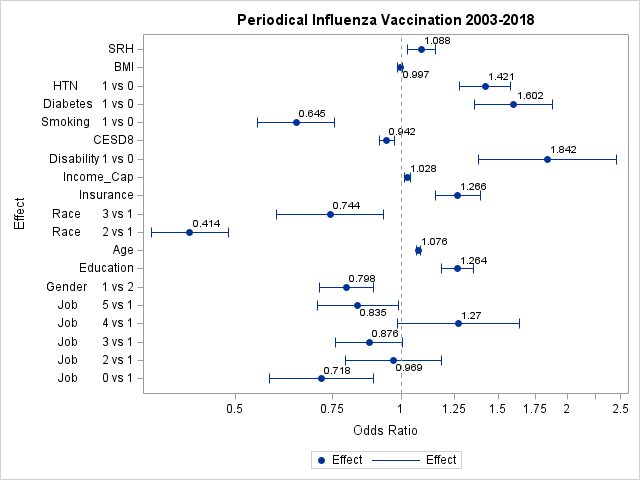


***Note.*** SRH=Self-rated health; BMI=Body mass index; HTN=hypertension; CES-D8=Center for Epidemiologic Studies Depression Scale-8 Items; Income_Cap=Household income per capita; Race: Others=3; Black=2. Gencer: men=1; women=2. Job Category (JOB): Management & Science=1; Social Services=2; General Services=3; Health Services=4; Industrial Workforce=5; Did not report job category=0.
